# Management of a cluster of *Clostridium difficile* infections among patients with osteoarticular infections

**DOI:** 10.1186/s13756-017-0181-4

**Published:** 2017-02-15

**Authors:** Jacqueline Färber, Sebastian Illiger, Fabian Berger, Barbara Gärtner, Lutz von Müller, Christoph H. Lohmann, Katja Bauer, Christina Grabau, Stefanie Zibolka, Dirk Schlüter, Gernot Geginat

**Affiliations:** 1 Institute of Medical Microbiology, Infection Control and Prevention, Otto-von-Guericke University of Magdeburg, Leipziger Straße 44, 39120 Magdeburg, Germany; 2 Department of Orthopedic Surgery, Otto-von-Guericke University of Magdeburg, Magdeburg, Germany; 3 Institute of Medical Microbiology and Hygiene, Consultant Laboratory for Clostridium difficile, University of Saarland, Saarland, Germany; 4 Institute for Laboratory Medicine, Microbiology and Hygiene, Christophorus Kliniken, Coesfeld, Germany; 5 Central pharmacy, Otto-von-Guericke University of Magdeburg, Magdeburg, Germany; 6 Organ-specific Immune Regulation, Helmholtz Centre for Infection Research, Braunschweig, Germany

**Keywords:** *C. difficile*, Ribotype 027, Rifampicin, Osteoarticular infections, Antibiotic stewardship

## Abstract

**Background:**

Here we describe a cluster of hospital-acquired *Clostridium difficile* infections (CDI) among 26 patients with osteoarticular infections. The aim of the study was to define the source of *C. difficile* and to evaluate the impact of general infection control measures and antibiotic stewardship on the incidence of CDI.

**Methods:**

Epidemiological analysis included typing of *C. difficile* strains and analysis of possible patient to patient transmission. Infection control measures comprised strict isolation of CDI patients, additional hand washings, and intensified environmental cleaning with sporicidal disinfection. In addition an antibiotic stewardship program was implemented in order to prevent the use of CDI high risk antimicrobials such as fluoroquinolones, clindamycin, and cephalosporins.

**Results:**

The majority of CDI (*n* = 15) were caused by *C. difficile* ribotype 027 (RT027). Most RT027 isolates (*n* = 9) showed high minimal inhibitory concentrations (MIC) for levofloxacin, clindamycin, and remarkably to rifampicin, which were all used for the treatment of osteoarticular infections. Epidemiological analysis, however, revealed no closer genetic relationship among the majority of RT027 isolates. The incidence of CDI was reduced only when a significant reduction in the use of fluoroquinolones (*p* = 0.006), third generation cephalosporins (*p* = 0.015), and clindamycin (*p* = 0.001) was achieved after implementation of an intensified antibiotic stewardship program which included a systematic review of all antibiotic prescriptions.

**Conclusion:**

The successful reduction of the CDI incidence demonstrates the importance of antibiotic stewardship programs focused on patients treated for osteoarticular infections.

## Background

*Clostridium difficile* is a gram-positive, anaerobic bacterium which is ubiquitously present in the gastrointestinal tract of humans and animals [1, 2]. As a spore-forming bacterium, *C. difficile* has the ability to persist and to remain infectious in the environment for extended periods of time. Spores are highly resistant to desiccation and alcohol disinfectants [1]. The main risk factors for development of *C. difficile* infection (CDI) are (i) previous antibiotic treatment, in particular with high risk antibiotics such as 3^rd^ generation cephalosporins, clindamycin, and fluoroquinolones, (ii) long hospitalization, (iii) underlying comorbidities, and (iv) high age of patients [1, 3]. Also specific risk factors for ribotype 027 (RT027) such as selective decontamination of the digestive tract and a longer length of stay in the ICU have been reported [4]. Despite it is well established that antimicrobial therapy with clindamycin and levofloxacin results in dysbiosis and enhanced risk to develop CDI, it is suspected that simultaneous therapy with rifampicin to some degree protects patients from CDI [5–7]. The increasing incidence of severe CDI among hospitalized patients is an enormous clinical problem [8, 9]. However, data on the CDI incidence among patients with prosthetic joint infections are scarce [10]. In addition, the CDI incidence among orthopedic patients after clean surgery such as primary arthroplasty of the hip or knee is very low (0.17%) [10]. A much higher CDI incidence of 7.1%, however, has been reported after open reduction and internal fixation of intertrochanteric femoral fractures, which in all cases occurred after previous antibiotic therapy [11].

Between June 2014 and December 2015 we observed an increased incidence of CDI among patients suffering from mostly implant-associated osteoarticular infections, where the majority of CDIs (57%) was caused by *C. difficile* RT027. The aims of this study were to define risk factors, possible sources of infection, and to evaluate the impact of general infection control measures and antibiotic stewardship on the incidence of CDI in this difficult to treat group of patients.

## Methods

### Patient population, CDI case definition, and analysis of risk-factors

The current study was initiated after recognition of a possible CDI outbreak in the first quarter 2015 on the septic ward of the department of orthopedic surgery. This department with three independent wards is part of an 1,100 bed tertiary care university hospital and acts as a regional referral center for the treatment of osteoarticular infections. Patients with osteoarticular infections stay on a specialized ward to which we refer as “septic ward”.

For the study hospital-acquired CDI was defined as new onset of diarrhea 48 h after hospital admission and laboratory-confirmed detection of *C. difficile* toxin genes by PCR. Severe CDI was defined by the presence of at least one of the following symptoms: fever >38.5 °C, decreased kidney function (creatinine >1.5x10^3^g/L) and/or high leukocyte count (>15 × 10^9^ cells/L). A recurrent CDI case was defined as new onset of CDI within 8 weeks after resolution of a previous CDI episode. No children were included in the study. An outbreak situation was defined as two or more CDI cases on a single ward within 28 days. Risk factors for infection with *C. difficile* RT027 were evaluated by comparing patients with CDI caused by RT027 with patients infected with non-RT027 ribotypes.

### Infection control and antibiotic stewardship

Infection control measures were implemented after recognition of an enhanced CDI incedence. The infection control bundle included (i) handwashing with soap after disinfection, to reduce first vegetative forms of *C. difficile* or other bacteria and secondly spores of *C. difficile*, (ii) use of hygienic bags for bedpans, (iii) strict isolation or cohorting of infected patients, (iv) personalized use of all materials with direct contact to the patient e.g. stethoscope, and (v) disinfection of surfaces and equipment in all wards, operating rooms and physiotherapy with peracetic acid and peroxide hydrogen vaporization after discharge of patients. Intensified environmental cleaning procedures were controlled by sampling of surfaces as described below. Additionally, health care workers were educated on diagnosis, treatment and prevention of CDI and provided with an antibiotic risk checklist which groups of antibiotics in 3 risk categories (low risk: linezolid, vancomycin, metronidazole, tetracycline, trimethoprim/sulfamethoxazole, fosfomycin; daptomycin, medium risk: all beta-lactam-antibiotics with the exception of third generation cephalosporins; high risk; clindamycin, third generation cephalosporins, fluoroquinolones). Physicians were instructed to follow the in-house guidelines for antibiotic therapy and microbiological diagnostic of osteoarticular infections and to avoid the prescription of high risk antibiotics for therapy. In quarter 3/2015 an intensified antibiotic stewardship program was implemented which included a weekly review of all antibiotic prescriptions by an orthopedic surgeon and a clinical microbiologist trained in antibiotic stewardship. The organization of the team was not changed since the beginning of the intervention. If possible antibiotic therapy was adjusted in order to avoid CDI high risk antibiotics as described above.

In order to control the impact of infection control measures and antibiotic stewardship the CDI incidence was monitored as number of cases per 1,000 hospital bed days per quarter.

In addition the impact of the antibiotic stewardship intervention was controlled by quarterly monitoring of antibiotic consumption on the septic ward. Antibiotic consumption was calculated as defined daily doses (DDD) per 100 hospital bed days according to WHO standards [12].

### Diagnostic specimens and CDI laboratory diagnostic testing

Diagnostic stool specimens were collected from 63 adult patients showing symptoms of diarrhea between June 2014 and December 2015. Stool diagnostic was performed as part of the routine microbiological diagnostic in a two-step algorithm.

First, clinical samples were primarily screened with a Clostridium glutamate dehydrogenase (GDH)-specific enzyme-linked immunosorbent assay (RIDASCREEN® *Clostridium difficile* GDH, R-Biopharm, Darmstadt, Germany) using the protocol provided by the manufacturer. In case of positive results (*n* = 29) DNA was extracted from the original stool sample. In brief, 100 μl stool in 900 μl sample buffer were used, followed by centrifugation at 1,000 x g for 5 min. Subsequently 400 μl supernatant was transferred into Precellys® Soil grinding SK38 tubes (Bertin Technologies, USA) and homogenized by centrifugation (5,000 x g for 75 sec) using the MagNA Lyser System (Roche Diagnostics, Mannheim, Germany). The lysate was clarified by centrifugation at 1,000 x g for 5 min and subsequent incubated for 10 min at 70 °C in a thermoshaker. DNA-extraction was performed using the QIAamp® DNA Mini Kit (Qiagen, Hilden, Germany) according to manufacturer’s instructions.

Second, purified DNA samples were tested for the presence of *C. difficile* DNA using two commercially available real-time PCR test systems. *C. difficile* toxins A (*tcdA*) and B (*tcdB*) genes were detected using the RealStar® *Clostridium difficile* PCR Kit 1.0 (altona Diagnostics, Hamburg, Germany). The binary toxin gene and the Δ117 deletion in the *tcdC* gene were detected using Xpert® *C. difficile*/Epi PCR assay (GeneXpert, Cepheid, Sunnyvale, CA, USA). All tests were performed according to manufacturer’s protocol.

### Multilocus sequence typing (MLST)

MLST was performed with DNA either isolated from feces (*n* = 10) or from isolated strains (*n* = 16). The MLST was performed as described before [13], targeting 7 housekeeping genes of *C. difficile*: *adk*, *atpA*, *dxr*, *glyA*, *recA*, sod and *tpi*. The sequencing reactions were run on a 3130xl Genetic Analyzer (Applied Biosystems). Editing, alignment, and phylogenetic analysis of sequences were performed with the program MEGA 6.0 [14]. DNA sequences were uploaded to the MLST database and *C. difficile* sequence types (ST) were received from the website [15].

### *Clostridium difficile* culture and susceptibility testing

*Clostridium difficile* was cultured form GDH-positive stools (*n* = 16) using a chromogenic medium (chromID™ *C. difficile*, bioMérieux, Marcy l’Etoile, France). The medium was inoculated with 10 μl feces and incubated anaerobically at 35 ± 1 °C for 7 days. Presumptive *C. difficile* colonies were confirmed by MALDI-TOF MS (VITEK® MS, bioMérieux).

The minimal inhibitory concentrations (MIC) of metronidazole, vancomycin, rifampicin, levofloxacin, and clindamycin were determined by gradient strip test (Etest, bioMérieux) on Brucella blood agar (Becton Dickinson, Heidelberg, Germany) inoculated with 100 μl solution of a 1.0 McFarland suspension of *C. difficile* in saline as described previously [16]. Agar plates were incubated under anaerobic conditions at 35 ± 1 °C for 48 h. For the interpretation of MIC the EUCAST epidemiological cut off values (ECOFF) were used for metronidazole (>2 mg/L), vancomycin (>2 mg/L), rifampicin (>0.004 mg/L). No ECOFF are available for clindamycin and levofloxacin.

### Enviromental sampling of inpatient environment

Sampling of the inpatient environment for the presence of *C. difficile* was performed as described [17]. Briefly, surface samples were taken using 25 cm^2^ sponge swabs pre-moistened with neutralizing solution (Lab M Ltd, Heywood, United Kingdom). Frequent contact surfaces of the patient room (head/foot-end boards of patient beds, bed rail, bedside table, nurse call button, patients telephone) and the en-suite bathroom (toilet seat, toilet assist handle) were sampled. After sampling sponge swabs were placed aseptically into the sterile sample transport bag prefilled with 10 ml neutralizing solution. The laboratory bags were opened and supplemented with 40 ml sterile phosphate-buffered saline (Becton Dickinson, Heidelberg, Germany) to yield a final volume of 50 ml. Sponge bags were resealed and homogenized manually by massaging the bag for 1 min. After 10 min. incubation at room temperature the whole volume was passed through a 45 μm membrane filter (Pall GmbH Laboratory, Dreieich, Germany). Filters were aseptically put onto Brazier's CCEY agar (Oxoid, Wesel, Germany) and incubated 48 h in an anaerobic atmosphere at 35 ± 1 °C. After 48 h, suspected *C. difficile* colonies were further analysed by MALDI-TOF MS. Confirmed *C. difficile* isolates were tested for toxin production and typed by MLST.

### Ribotyping and multiple-locus variable-number tandem repeat analysis (MLVA)

Ribotyping and MLVA analysis of 11 ST1 isolates was performed by the National Consultant Laboratory for *C. difficile* in Homburg/Saar (University of Saarland Medical Center, Homburg, Germany). PCR-ribotyping was performed for each isolate according the standard protocol (European harmonized diagnostic procedures ECDIS; http://www.ecdisnet.eu) as described earlier [18] which included capillary gel electrophoresis of fluorescent labelled fragments (Beckmann Coulter, Brea, California USA) and ribotype assignment to an institutional databank by an automated software tool (BioNumerics version 7.1, Applied Math, Sint-Martens-Latem, Belgium). MLVA was carried out as described previously [19] with BioNumerics as automated software (version 7.1, Applied Math, Sint-Martens-Latem, Belgium). The definition of clonality was based upon previous studies with a genetic difference of less than 3 repeats while a clonal cluster was defined by ≤2 repeat differences and genetic related isolates by ≤10 repeat differences [20].

### Statistical analysis

Statistical analyses were performed using the Fisher’s exact Test for bivariate analysis of *C. difficile* risk factors (non RT027 CDI versus RT027 associated CDI) and the two-sample independent *t* Test for the means of antibiotic consumption data before and after intervention with a significance level of *p* values <0.05. The Microsoft Excel statistic tool, OpenEpi (http://www.openepi.com) was used to analyze the data. The upper and lower statistical boundaries were defined as the mean CDI incidence of the four previous quarters plus/minus standard deviation (MA ± SD).

## Results

### Clustering of CDI cases

Retrospective analysis showed that already in the third quarter of 2014 the CDI incidence (1.41 cases per 1,000 hospital bed days) on the septic ward, a special unit for treatment of patients with osteoarticular infections was significantly above average incidence of the whole department (0.64 cases per 1,000 hospital bed days, *p* = 0.036). Orthopedic infections in our patient cohort were associated with endoprosthesis (19/26; 65.5%), soft tissue/wound infections (4/26; 15.4%), septic spondylitis (2/26; 7.7%), and osteomyelitis (1/26; 3.8%).

**Fig. 1:**
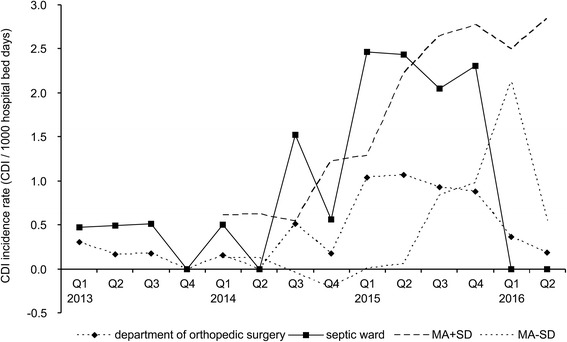
Incidence of *C. difficile* infections during the study period. The CDI incidence rates (number of cases per 1000 hospital bed days per quarter) for the whole department of orthopedic surgery (diamonds) and the septic ward only (squares) are shown. For every quarter the moving average (MA) of the previous four quarters was calculated. For CDI surveillance upper (MA + SD, upper dotted line) and lower (MA-SD, lower dotted line) boundaries were defined. An increase or decrease of the CDI incidence was considered significant if the actual CDI incidence crossed the upper or lower boundaries, respectively

In quarter 3/2014 the CDI incidence was above the mean CDI incidence of the 4 previous quarters plus SD (Fig. 1). The mean CDI incidence on the septic ward increased significantly from 0.33 infections per 1,000 hospital bed days (SD 0.25) in the period from quarter 1/2013 to 2/2014 to 2.3 infections per 1,000 hospital bed days (SD 0.98) in quarters 1/2015 to 4/2015 (*p* = 7.2 x 10^−7^). After implementation of an intensified antibiotic stewardship program in quarter 3/2015 the CDI incidence dropped in the first quarter 2016 to no case per quarter which was significantly below the mean CDI incidence of the 4 previous quarters minus SD of 2.13 infections per 1,000 hospital bed days (Fig. 1). Also in the second quarter 2016 no CDI case was monitored.

From June 1st 2014 to December 31th 2015 a total number of 63 patients with gastrointestinal disorders were tested for the presence of *C. difficile*. GDH-positive samples from 29 patients were further tested for the presence of toxin genes by PCR. CDI was confirmed by PCR in 26 patients, which also demonstrated the clinical symptoms of CDI. With exception of two samples, sequence types were determined by MLST from toxin gene-positive samples, 15 samples (57.7%) were typed as ST1 and were further identified as RT027 by CE-ribotyping. The following sequence types were identified: ST3 (*n* = 2), ST6 (*n* = 2), ST14 (*n* = 2), ST8 (*n* = 1), ST15 (*n* = 1), and ST92 (*n* = 1). In all samples both toxin encoding genes were detectable, whereas the binary toxin gene and the Δ117 in the *tcdC* gene were only present in samples typed as ST1.

The time between admission of patients to the septic ward and diagnosis of CDI ranged from 2 to 72 days. Characteristics and co-morbidities of patients were summarized from patients records (Table 1). Renal insufficiency, treatment with gastric acid suppressors and treatment with CDI high risk antibiotics were the most frequent risk factors but were not significantly different among patients infected with *C. difficile* RT027 (ST1) and non-RT027 strains. The only significant risk factor for CDI caused by RT027 was a patient’s stay on the septic ward.

**Table 1:** Description of study cohort and assessment of potential risk factors for CDI

		CDI cases	Non-RT027	RT027	*p* values^a^
Median age		73.08 (range 54–87)	71.63	74.13	
Sex	female	16	6	10	0.41
	male	10	5	5	0.41
Hospitalization on septic/non-septic ward	septic ward	21	7^d^	14	0.04
	all other wards	5	4 ^b,c^	1	
Primary diagnosis	periprosthetic infection	19	8	11	0.81
	soft tissue / wound infection	4	1	3	
	spondylitis	2	2	0	
	osteomyelitis	1	0	1	
Co-morbidities	renal insufficiency	12	4	8	0.41
	diabetes	11	5	6	
	pneumonia	1	0	1	
	neoplasm	2	1	1	
	hepatic disease	2	1	1	
Antimicrobial therapy	aminopenicillins	4	1	3	0.72
	ureidopenicillins	6	2	4	0.42
	fluoroquinolones	9	3	6	0.27
	cephalosporins	13	5	8	0.24
	clindamycin	7	3	4	0.81
	rifampicin	13	6	7	0.69
Gastric acid suppressors	proton pump inhibitors/H2 blockers	25	11	14	
Severity factors	creatinine (>1.5x10^3^g/L)	7	3	4	0.99
	leukocytosis (>1.5x10^9^/L)	10	4	6	0.93
Antimicrobial CDI treatment					
First episode	metronidazole po.	18	8	10	
	vancomycin po.	1	0	1	
	metronidazole iv. vancomycin po.	2	unknown	unknown	
	fidaxomicin po.	4	1	3	
	colectomy	1	0	1	
	no treatment	1	0	1	
Recurrence	vancomycin po.	5	2	3	
	metronidazole iv. + vancomycin po.	1	0	1	
	fidaxomicin po.	3	1	2	
	fecal biota transplantation	1	0	1	
Clinical outcome	cure	15	9	6	0.21
	recurrence	10	3	7	0.37
	death within 6 months	4	0	4	0.13
	death CDI attributed	1	0	1	
	death CDI contributed	3	0	3	

^a^ *p* values were determined with Fisher’s exact Test

^b^ Sequence type ST 6

^c^ Sequence types ST3, 14, 15

^d^ Sequence types ST3, 6, 8, 14, 92, and two unknown strains

### Outcome of CDI

Non-severe CDI was initially diagnosed in 18 from 26 patients (69.2%) and in 6 from 15 patients (40.0%) infected with RT027. Treatment of CDI was initiated in all cases according to current European Society of Clinical Microbiology and Infectious Diseases guidelines [21]. After primary CDI treatment 15 (57.7%) patients recovered, among them 6 (40%) patients with RT027. Recurrences were observed in 10 (38.5%) patients. The mortality of 15.4% (4/26) within 6 months after diagnosis of CDI was exclusively attributable to CDI due to *C. difficile* RT027.

### Infection control and antibiotic stewardship

After clustering of CDI cases was first recognized in the quarter 1/2015 a primary infection control and antibiotic stewardship bundle was implemented (see methods section).

Orthopedic infections generally require a long-term (≥6 weeks) antibiotic treatment [22–24]. In most cases, antibiotic therapy of the primary orthopedic infection consisted of two or more antibiotics. The most frequently prescribed classes of antibiotics were fluoroquinolones (34.6%), cephalosporins (50.0%), clindamycin (26.9%), rifampicin (50.0%), and the penicillin/betalactamase inhibitor combinations ampicillin/clavulanic acid (15.4%) and piperacillin/tazobactam (23.1%) (Table 1).

Initial review of antibiotic prescription data indicated an overuse of CDI high risk antibiotics (Table 2) although an in-house guideline for antibiotic therapy and infectious disease diagnostics had been in use for several years. The primary antibiotic stewardship intervention focused on information of medical doctors, who were provided with an antibiotic risk checklist which groups of antibiotics in 3 risk categories. Physicians were instructed to follow the in-house guidelines for antibiotic therapy and microbiological diagnostic of osteoarticular infections and to avoid the prescription of high risk antibiotics for therapy. An intensified antibiotic stewardship program was implemented during quarter 3/2015 which included a weekly review of all antibiotic prescriptions by a clinical microbiologist.

**Table 2:** Antibiotic consumption in the septic ward during quarters 1/2013 to 2/2016

	Antibiotic consumption (DDD/100 hospital bed days)														*p* ^a^
	2013				2014				2015				2016		
	Q1	Q2	Q3	Q4	Q1	Q2	Q3	Q4	Q1	Q2	Q3	Q4	Q1	Q2	
Narrow spectrum penicillins^b^	8.8	20.8	16.5	26.3	6.8	11.3	8.3	15.0	26.5	19.6	13.0	34.8	35.0	52.0	0.131
Amino penicillin/BLI	7.8	3.2	0.5	1.8	6.4	4.1	15.4	4.3	7.2	10.9	16.7	16.3	10.0	7.2	0.044
Broad spectrum penicillins^c^	2.7	1.0	1.6	0.0	2.4	3.4	5.2	2.0	4.5	2.3	8.2	4.5	3.5	5.1	0.046
Cephalosprins 1^st^ and 2^nd^ gen.	12.2	27.2	42.3	32.3	35.5	43.3	23.0	30.5	21.5	32.8	22.2	23.0	14.0	23.5	0.016
Cephalosprins 3^rd^ gen.	2.1	3.2	2.1	2.4	4.1	3.7	0.8	1.5	0.9	0.6	1.7	0.0	1.8	0.0	0.015
Carbapenems	4.8	3.2	5.1	2.7	7.9	3.0	4.9	5.9	3.4	5.0	5.1	10.0	2.4	6.8	0.283
Fluoroquinolones	49.7	35.0	29.7	21.6	32.6	45.1	34.3	32.0	33.6	31.2	26.0	6.6	4.3	10.6	0.006
Clindamycin	9.1	12.1	19.3	0.0	12.3	25.2	19.4	12.6	22.1	15.2	6.8	3.7	4.4	5.1	0.001
Linezolid	2.4	3.2	4.4	2.4	4.0	3.6	3.5	2.0	2.8	7.9	4.8	6.9	8.7	10.1	0.019
Glycopeptide/daptomycin	5.4	5.9	5.7	1.5	6.2	4.5	4.6	3.8	5.1	3.6	4.8	8.9	5.4	13.8	0.143
Rifampicin	18.3	14.1	19.2	0.4	9.7	9.9	0.0	0.0	3.5	10.1	4.5	17.8	12.2	13.9	0.243
Total	125.6	132.3	150.6	106.9	129.7	157.3	122.7	113.1	133.0	141.6	122.1	133.5	103.5	150.2	0.348

^a^Comparision of average antibiotic consumption before (Q1/213-Q2/2015) and after (Q3/2105-Q2/2016) implementation of an intensified antibiotic stewardship program. ^b^narrow spectrum penicliins: penicillin G, flucloxacillin, aminopenicillins, ^c^ broad spectrum penicillins: piperacillin, piperacillin/tazobactam aminopenicillins

The quarterly analysis of antibiotic consumption between quarters 1/2013 and 2/2016 shows that fluoroquinolones, 1^st^ and 2^nd^ generation cephalosporins and clindamycin were the most prescribed antibiotics on the septic ward, even after the initial recommendation to avoid these antibiotics after recognition of the enhanced CDI incidence in quarter 1/2015 (Table 2).

**Fig. 2:**
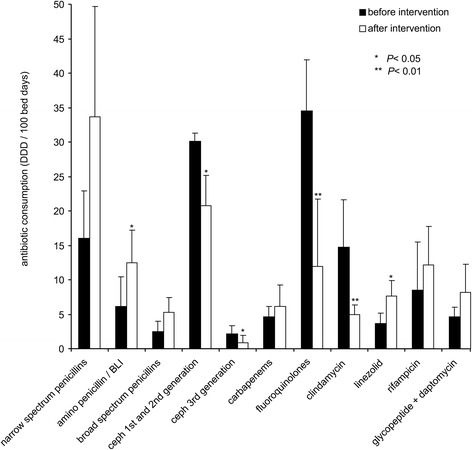
Antibiotic prescription for patients of the septic ward before and after implementation of the intensified antibiotic stewardship program in quarter 3/2015. Bars indicate the mean antibiotic consumption calculated as defined daily doses (DDD) per 100 hospital bed days. For the major classes of antibiotics the mean antibiotic consumption and standard deviations were calculated for quarters 1/2013 to 2/2015 before intervention and quarters 3/2015 to 2/2016 after intervention. Narrow spectrum penicillins include benzylpenicillin, flucloxacillin, and aminopenicillins; broad spectrum penicllins include piperacillin and piperacillin/tazobactam. Asterisks indicate a significant (*p* values <0.05) difference of antibiotic consumption before and after intervention

Significant reduction of antibiotic consumption of *C. difficile* high risk antibiotics was achieved after the intensified antibiotic stewardship program for septic orthopedic patients was initiated in quarter 3/2015. This intervention led to a significant reduction (*p* values <0.05, Table 2, Fig. 2) of the consumption of fluoroquinolones, clindamycin, and 1st and 2^nd^ generation cephalosporins and increased use of narrow spectrum penicillins (benzylpenicillin and flucloxacillin, +110%), linezolid (+111%), and rifampicin (+41%). The total average antibiotic consumption before intervention was 131.3 DDD per 100 hospital bed days and after intervention 127.3 DDD per 100 hospital bed days, respectively. Thus, although consumption of high risk antimicrobials was significantly lowered total antibiotic consumption remained on a high level after the intervention.

### Antibiotic susceptibility testing of *C. difficile* isolates

In vitro antibiotic susceptibility tests were performed with 16 *C. difficile* isolates (ST1 (*n* = 11), ST6 (*n* = 2), ST8 (*n* = 1), ST14 (*n* = 1), and ST92 (*n* = 1) (Table 3). All ST1 (RT027) isolates showed an elevated MIC for multiple antibiotics including levofloxacin (MIC ≥32 mg/L), and rifampicin (MIC ≥32 mg/L). Also the MIC of clindamycin was elevated among the majority of RT027 isolates. All non-RT027 strains showed a low MIC for rifampicin (<0.002 mg/L) and the MIC of clindamycin (2.0–8.0 mg/L) was under the epidemiological cut-off value of 16.0 mg/L. The MIC of levofloxacin was 6.0 mg/L (ECOFF not available).

**Table 3:** MLST types and MICs for selected antibiotics of *C. difficile* isolates

Inpatient #	MLST type	RT	MIC (mg/L)				
			Metronidazole	Vancomycin	Rifampicin	Levofloxacin	Clindamycin
1	ST1	027	1	1.5	≥32	≥32	≥256
2	ST1	027	1	1.5	≥32	≥32	≥256
3	ST1	027	1	1.5	≥32	≥32	6.0
4	ST1	027	1	1.5	≥32	≥32	2.0
5	ST1	027	1	1.5	≥32	≥32	≥256
6	ST1	027	1	1.5	≥32	≥32	≥256
7	ST1	027	1	1.5	≥32	≥32	≥256
8	ST1	027	1	1.5	≥32	≥32	≥256
9	ST1	027	1	1.5	≥32	≥32	≥256
10	ST1	027	1	1.5	≥32	≥32	≥256
11	ST1	027	1	1.5	>32	>32	≥256
12	ST1	n.d.	n.d.	n.d.	n.d.	n.d.	n.d.
13	ST1	n.d.	n.d.	n.d.	n.d.	n.d.	n.d.
14	ST1	n.d.	n.d.	n.d.	n.d.	n.d.	n.d.
15	ST1	n.d.	n.d.	n.d.	n.d.	n.d.	n.d.
16	ST3	n.d.	n.d.	n.d.	n.d.	n.d.	n.d.
17	ST3	n.d.	n.d.	n.d.	n.d.	n.d.	n.d.
18	ST6	n.d.	0.25	0.05	<0.002	6.0	3.0
19	ST6	n.d.	0.38	0.05	<0.002	6.0	8.0
20	ST8	n.d.	0.25	0.05	<0.002	6.0	2.0
21	ST14	n.d.	n.d.	n.d.	n.d.	n.d.	n.d.
22	ST14	n.d.	0.5	0.5	<0.002	6.0	2.0
23	ST15	n.d.	n.d.	n.d.	n.d.	n.d.	n.d.
24	ST92	n.d.	0.125	0.5	<0.002	6.0	8.0
25	unknown	n.d.	n.d.	n.d.	n.d.	n.d.	n.d.
26	unknown	n.d.	n.d.	n.d.	n.d.	n.d.	n.d.

n.d. not determined

### Epidemiological analysis

**Fig. 3:**
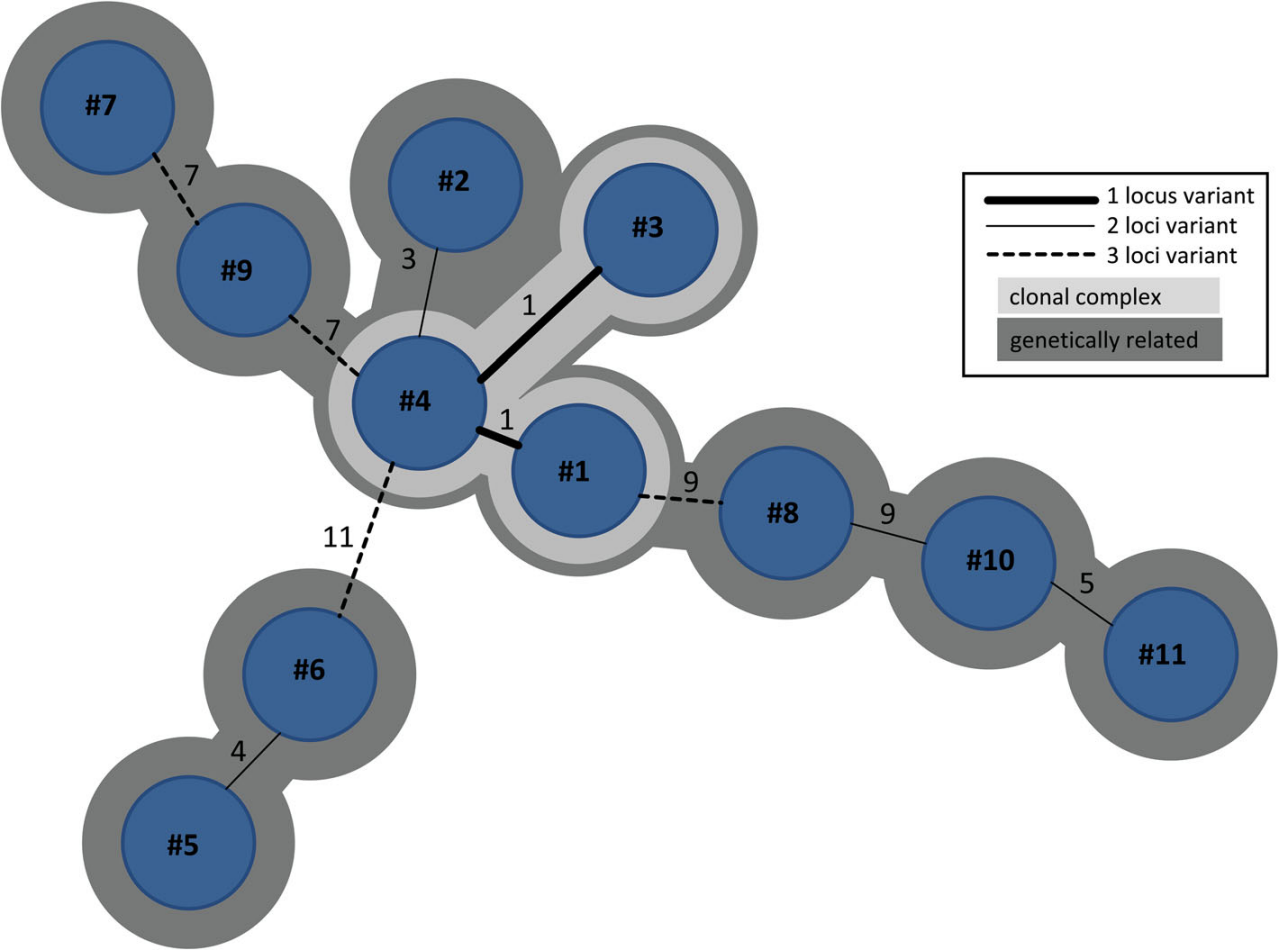
MLVA minimum spanning tree of 11 RT027 isolates

**Fig. 4:**
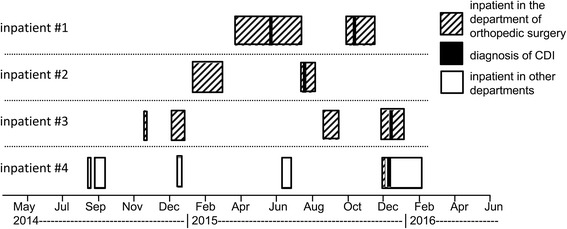
Admission and discharge data (month and year) of CDI cases from which genetically related *C. difficile* were isolated

The inquiry by infection control personal yielded no noticeable epidemiological association among patients with identical non-RT027 strains (data not shown). In order to confirm a possible epidemiological association of RT027 isolates form the septic ward subtyping by MLVA was performed. The typing of 11 RT027 isolates revealed two genetically related clusters (defined as ≤10 repeat differences). The larger cluster comprised 9 isolates (isolates 1–4 and 7–11) and the minor cluster 2 isolates (isolates 5 and 6) (Fig. 3). Only three isolates (1, 3, and 4) showed clonal relatedness (defined as ≤2 repeat differences). A fourth isolate (# 3) showing three repeat differences was also closely related to this cluster. We further analyzed the possible epidemiological association of the four patients from which these genetically closely related strains (repeat differences ≤3) were isolated. A detailed inquiry of possible contacts, however, did not reveal any direct or indirect contacts of inpatients infected within these related RT027 isolates. As shown in Fig. 4, there were no overlaps of admission and discharge data of excluding direct contact.

In order to further exclude a remnant reservoir of viable *C. difficile* RT027 in patient rooms after discharge of the patient and cleaning of the room, environmental investigations of surfaces of patient rooms were performed. Among 60 environmental samples taken in August 2015, December 2015, and January 2016 *C. difficile* RT027 was isolated from only one sample from the telephone set of an inpatient infected with RT027, without any transmissions of this particular strain to other inpatients up to now. Thus indicating that cleaning procedures were adequate and a persistent environmental *C. difficile* reservoir in patient rooms was highly unlikely.

## Discussion

Here we report an increased incidence of CDI mainly caused by *C. difficile* RT027 with an elevated MIC for rifampicin among patients suffering from osteoarticular infections and the successful control of CDI by implementation of an antibiotic stewardship program focused on this difficult to treat group of patients.

The dominance of *C. difficile* RT027 among patients with orthopedic infections observed in the current study could be due to a number of different reasons. During the primary outbreak analysis, the investigation focused on a possible common source of *C. difficile* RT027 isolates. The only risk factor for CDI caused by RT027 was a patient’s stay on the septic ward neither molecular epidemiological analysis, nor analysis of inpatient contacts, nor environmental sampling suggested a possible common source of RT027 isolates. Probably, CDI was mainly restricted to the septic ward due to the higher exposure of inpatients to CDI high risk antibiotics required for the treatment of osteoarticular infections. Antibiotic consumption of high risk antibiotics on the septic ward was 2 to 3-fold higher compared to the other wards of the orthopedic department (data not shown). The majority of complications was associated with RT027, however due to the low number of patients the observed differences between patients infected with RT027 and non-RT027 were not significant.

The bundle of optimized antibiotic treatment, environmental cleaning procedures and education of health care workers effectively controlled nosocomial CDI thus corroborating previously published studies [10, 25–27]. Implementation of infection control measures alone, however, did not reduce the incidence of CDI, which was only achieved after drastic reduction of the consumption of *C. difficile* high risk antibiotics. The incidence of CDI was significantly reduced only after implementation of an intensified antibiotic stewardship program which lowered the prescription of fluoroquinolones, clindamycin, and cephalosporins in favor of low risks antibiotics such as penicillins and linezolid.

Average total antibiotic consumption before intervention was 131 DDD/100 hospital bed days and after intervention 127 DDD/100 hospital bed days. The antibiotic stewardship intervention shifted antibiotic therapy towards the use of narrow spectrum penicillins (benzylpenicillin and flucloxacillin, +110%), linezolid (+111%), and rifampicin (+41%). The DDD of benzylpenicillin is 3.6 g. Treatment of serious infections, however, generally requires a dose of 12 g or even higher. Thus calculating DDD while using much higher therapeutic doses results in an overestimation of the prescribed doses [28]. If correcting this by using recommended daily doses (RDD) [28] for the calculation of antibiotic consumption the antibiotic consumption before intervention was 99.0 RDD/100 hospital bed days and after intervention 86.1 RDD/100 hospital bed days, indicating that the number of prescribed doses on the septic ward was reduced roughly 13%. Total antibiotic consumption on the septic ward, however, remained significantly above the available national benchmark data for surgical departments other than general surgery (75% quantile 56 RDD/100 hospital bed days) [28, 29]. To our knowledge no benchmark data are available for units specialized on septic orthopedic surgery like the septic ward studied here. On the septic ward inpatients often require high-dose antibiotic combination therapies which explain the significantly higher antibiotic consumption compared to general orthopedic departments.

The average consumption of fluoroquinolones was 34.5 DDD (33.3 RDD) per 100 hospital bed days before and 11.5 DDD (10.3 RDD) per 100 hospital bed days after implementation of intensified antibiotic stewardship program. Despite after the intervention roughly 70% less quinolones were used overall quinolone consumption was still significantly above the national benchmark (75% quantile 6.9 RDD/100 patient days) [28, 29]. The consumption of first and second generation cephalosporins was reduced roughly 30% by replacing them with flucloxacillin for the therapy of susceptible staphylococci and by avoiding prolonged perioperative prophylaxis.

In Germany regionally between 5.3% and 33.5% of CDI are caused by RT027 [30, 31]. In our study 66% of CDI on the septic ward were caused by RT027, suggesting selection of this particular strain. Because for the study region no data are available for the rate of asymptomatic RT027 carriers, we cannot judge the degree of selection of RT027 among our patient group.

Antimicrobial therapy of osteoarticular infections often requires prolonged antibiotic treatment for 6 to 12 weeks [22–24]. According to current guidelines an initial parenteral therapy with beta-lactam antibiotics is often followed by oral therapy with antibiotics such as fluoroquinolones and rifampicin with good bioavailability, effective tissue and bone penetration, and biofilm activity [22–24]. Nevertheless, reports indicating an enhanced CDI incidence among patients with prosthetic joint infections are scarce [10]. Our current working hypothesis is that cephalosporins, quinolones and clindamycin as high risk antibiotics trigger CDI and concurrent treatment with rifampicin protects patients from CDI caused by rifampicin-susceptible *C. difficile*. Thus the reduced susceptibility of RT027 to rifampicin might increase the risk of CDI among patients treated with rifampicin [5, 32, 33]. However, rifampicin alone probably did not trigger CDI because less prescription of high risk antimicrobials even without reduction of the consumption of rifampicin significantly lowered the CDI incidence. The *ClosER* (*Clostridium difficile* European Resistance) study reported elevated MICs of *C. difficile* for fluoroquinolones and clindamycin among various ribotypes, whereas elevated rifampicin MICs were mainly associated with a few ribotypes such as RT027, RT018, and RT356 which are more common in South Eastern Europe [34]. It has also been shown before that long-term rifampicin treatment may result in the selection of *C. difficile* strains with an elevated rifampicin MIC [32–34]. In view of the low rifampicin MIC of the majority of *C. difficile* strains, it has been hypothesized that rifampicin might prevent CDI in osteoarticular infections [5–7].

Our study has a number of limitations. Firstly, because culture-based diagnostic of *C. difficile* was initiated by the laboratory after recognition of the epidemic situation in May 2015 the initial epidemiological analysis was performed based on PCR and MLST only. Sequence type 1 (ST1) can correspond to several ribotypes in particular RT176 [2], and the GeneXpert cannot differentiate between RT027 and RT176 [35, 36]. In Germany, however, RT176 is rather uncommon [37, 38], and ribotyping of 11 isolates confirmed that all ST1 belong to RT027. Because the discriminatory power of MLST alone is not sufficient for the analysis of the molecular epidemiology of *C. difficile* in outbreaks [39] MLVA analysis of 11 RT027 isolates was performed. The results indicated the presence of a very closely related strain in only four out of 11 patients, making a clonal outbreak caused by a common source in the hospital unlikely.

Secondly, the initially increased incidence of CDI in quarter 3/2014 correlated with the introduction of a new diagnostic test for the detection of *C. difficile*, which was switched from a toxin-specific enzyme-linked immunosorbent assay to primary screening with a GDH-specific ELISA followed by a toxin gen-specific PCR test. Thus, initially the increased number of patients with positive *C. difficile* tests was interpreted as result of the improved sensitivity of the new diagnostic test. Gould et al. reported an increase of the CDI incidence ranging from 43% to 67% after switching from toxin enzyme immunoassays to PCR-based *C. difficile* diagnostic tests [40]. Beginning with the third quarter 2014 the incidence of recurrent and severe CDI increased compared to the period before. Among all cases of CDI 28.6% of patients and among the cases of CDI caused by RT027 38,5% of patients had at least one criterion of severe CDI at the time point of laboratory diagnosis. In accordance with reported data, the CDI-associated mortality rate within 6 months was 15.4% for all CDI cases and 26.7% for RT027 cases, respectively. Severe CDI occurred mostly among elderly patients with multiple co-morbidities [10, 25, 27, 34, 41].

Third, despite the analysis of patient to patient contacts did not reveal a direct connection of our patients from which highly related RT027 were isolated we cannot strictly exclude a possible epidemiological connection. We could not exclude possible patient contacts outside the hospital and environmental screening was performed after cleaning and, therefore, the environment cannot be strictly excluded as source of infection. Also, screening of health care workers and asymptomatic patients was not performed which have been reported to act as vector for nosocomial transmission of *C. difficile* [42, 43].

## Conclusion

To our knowledge, this is the first report of an enhanced incidence of CDI caused by *C. difficile* RT027 with elevated MICs for rifampicin among patients with osteoarticular infections. The successful reduction of the CDI incidence demonstrates the importance of antibiotic stewardship programs focused on patients treated for osteoarticular infections. The impact of antibiotic stewardship on the restriction of *C. difficile* high risk antimicrobials should be tightly monitored in order to ensure that antibiotic stewardship recommendations are followed.

## Abbreviations

CDI: *Clostridium difficile* infection
*Clos*ER: *Clostridium difficile* European resistance study
DDD: Defined daily doses
ECOFFs: Epidemiological cut-off value
ELISA: Enzyme-linked immunosorbent assay
GDH: Clostridium glutamate dehydrogenase
MA+/− SD: Moving average plus/minus standard deviation
MIC: Minimum inhibitory concentration
MLST: Multilocus sequence typing
MLVA: Multiple locus variable-number tandem repeat analysis
n.d.: Not determined
Q1-4: Quarter 1–4
RT027: *C. difficile* ribotype 027
ST: Sequence type
WHO: World Health Organization
